# Outcomes of COVID-19 During the First Wave in Saudi Arabia: An Observational Study of ICU Cases from a Single Hospital

**DOI:** 10.3390/jcm14061915

**Published:** 2025-03-12

**Authors:** Saeed A. Alqahtani, Talal M. Alshammari, Eidan M. Alzahrani, Abeer A. Alaohali, Jaber S. Alqahtani, Yahya A. Alzahrani, Ahmad A. Alrawashdeh, Brett Williams, Mohammed A. Aljumaan, Amal H. Alsulaibikh, Mohannad A. Alghamdi, Mohammed A. Almulhim, Shaya Y. Alqahtani, Sarah Al-Ahmadi, Mohammed S. Alshahrani

**Affiliations:** 1 Department of Emergency Medical Care, Prince Sultan Military College of Health Sciences, Dhahran 34313, Saudi Arabiaalqahtani-jaber@hotmail.com (J.S.A.);; 2 Department of Emergency Medical Care, College of Applied Medical Sciences, Imam Abdulrahman Bin Faisal University, Dammam 34212, Saudi Arabia; 3 Department of Paramedics, Faculty of Allied Medical Sciences, Jordan University of Science and Technology, Ar-Ramtha 3030, Jordan; 4 Department of Paramedicine, Faculty of Medicine, Nursing, and Health Sciences, Monash University, Clayton, VIC 3168, Australia; 5 Department of Emergency Medicine, College of Medicine, Imam Abdulrahman Bin Faisal University, Dammam 34212, Saudi Arabia; mohaghamdi@iau.edu.sa (M.A.A.);; 6 Department of Intensive Care, King Fahad Hospital of the University of Imam Abdulrahman bin Faisal, Dammam 34212, Saudi Arabia; 7 Department of Internal and Critical Care Medicine, College of Medicine, Imam Abdulrahman Bin Faisal University, Dammam 34212, Saudi Arabia

**Keywords:** COVID-19, critical care, Middle East, Saudi Arabia, mortality

## Abstract

**Background/Objectives**: Mortality from COVID-19 in intensive care units (ICUs) was not clearly reported in many regions during the first wave. We aimed to assess the characteristics and outcomes of ICU patients with COVID-19 in Saudi Arabia. **Methods**: This was a secondary data analysis of the Convalescent Plasma Trial. All patients who were recruited from King Fahad Hospital of the University (KFHU) in the Eastern Region of Saudi Arabia between 13 March 2020 and 13 September 2020 were included. Characteristics and outcomes, differences in characteristics and outcomes between Saudi and non-Saudi populations, and predictors of mortality were assessed. **Results**: The KFHU recruited 185 ICU patients with COVID-19. Of those, 90 (48.6%) were Saudi, and 95 (51.4%) were non-Saudi. The overall mean age was 56.7 years, and 71.9% were males. Compared with Saudis, non-Saudis were younger, with a mean age of 54.4 years, were more likely to be males (81.1%), and had a higher median respiratory rate (28.0 breaths/min vs. 24.0), a lower percentage of blood-oxygen saturation (86.0% vs. 91.0%), and higher median levels of ferritin per µg/L (820 vs. 550). The overall mortality rate was 33.0% (*n* = 61). The mortality rate in non-Saudis (42.1%) was higher than that in Saudis (23.3%). The variables associated with increased mortality included non-Saudi status (odds ratio [OR] 2.66; 95% CI: 1.05, 6.72), ferritin (OR 1.01; 95% CI: 1.00, 1.02), and intubation (OR 8.55; 95% CI: 2.92, 24.97). **Conclusions**: Since the mortality rate in non-Saudis was greater than that in Saudis, more efforts should be made to improve social determinants of health across non-Saudis in our region.

## 1. Introduction

In December 2019, coronavirus disease 2019 (COVID-19) was first recognized in Wuhan, China [1]. It is caused by severe acute respiratory syndrome coronavirus-2 and is contagious in humans [2]. In March 2020, the World Health Organization characterized COVID-19 as a global pandemic because the number of affected people exceeded 110,000 across 114 countries with a case fatality of more than 4000 [3]. Despite this pandemic alarm, the average number of new cases and cases of death since then and up to June 2023 was approximately 578,000 and 5700 per day, respectively. Extrapolating these figures would lead to a global mortality rate of 1.0% [4]. However, this rate could be as high as 78.0–84.6% in a subgroup of the population, those who experienced severe symptoms of COVID-19 during the initial phase of the pandemic [5,6,7].

Evidence suggests that elevated levels of pro-inflammatory cytokines and dysregulated immune response were the major determinants of COVID-19 severity [8,9]. Patients with COVID-19 who present with pathological cytokine storm required treatment in the intensive care unit (ICU) [10]. The rate of mortality in patients with COVID-19 who were admitted to the intensive care unit (ICU) was estimated to be 41.7% during the initial phase of the pandemic, with significant variation across the studied regions [11]. In Asia, the reported mortality rate was 35.3%, whereas it was 42.0% in North America and 48.4% in Europe. Part of this variation could be related to differences in patient demographics, including age, ethnicity, and comorbidities, and part could be related to differences in management and treatment interventions. Importantly, this variation could be related to publication bias. Unfortunately, mortality-related data related to COVID-19 in the ICU were not clearly reported in many regions during the early phase of the pandemic. This lack of information may mask other unknown factors.

Since Saudi Arabia is part of the Eastern Mediterranean region and studies from this region were lacking, this study aimed to describe the characteristics and outcomes of patients who were admitted to the ICU with COVID-19 in Saudi Arabia during the first wave of the pandemic. Differences in characteristics and outcomes across Saudi and non-Saudi populations and predictors of mortality will also be assessed.

## 2. Materials and Methods

### 2.1. Study Design

This was a secondary data analysis of a subpopulation from the Convalescent Plasma Trial [12]. The trial methodology has been described in detail elsewhere [13]. Briefly, this was a multicenter, open-label, two-arm national trial. The aim of the trial was to assess the feasibility, safety, and efficacy of convalescent plasma in treating ICU patients with COVID-19 in Saudi Arabia. Twenty-two hospitals across Saudi Arabia participated in patient recruitment. Between the 13th of March 2020 and the 13th of September 2020, investigators from King Fahad Hospital of the University (KFHU) recruited 185 patients after providing written informed consent from all patients or their proxies. The hospital is a teaching healthcare facility and is affiliated with Imam Abdulrahman bin Faisal University (IAU). For the purpose of our study, all patients recruited from KFHU (*n* = 185) were included, and the data we obtained were deidentified. The access date for the study population data was 26 June 2022. Our study has ethical approval from the IAU (Approval number: 2022-01-253; Date: 23 June 2022).

### 2.2. Settings

The hospital is located in Al-Khobar, a city in the Eastern Region of Saudi Arabia. It services a population of more than 450,000 residents, of whom approximately 51.0% are non-Saudis, across a geographical area of 571 km^2^ [14]. The hospital provides a range of medical services, including emergency and intensive care, and has a capacity of 550 beds, 42 of which are allocated to the ICU [15]. The ICU is staffed with a multidisciplinary team of national and international board-certified physicians, including intensivists, pulmonologists, gastroenterologists, cardiologists, neurologists, and hematologists. It is also staffed with critical care nurses and respiratory care therapists.

### 2.3. Populations, Definitions, and Data Sources

Per the trial protocol, a participant was recruited if the participant was aged ≥18 years and had a confirmed case of COVID-19 and met the criteria for admission to the ICU [13]. A case was considered COVID-19 if it tested positive via real-time reverse transcription polymerase chain reaction (rRT–PCR). The criteria for ICU admission included dyspnea, a respiratory rate of ≥30 breaths/min, a percentage of blood oxygen saturation (SpO_2_%) of ≤93%, an arterial-oxygen partial pressure to inspired-oxygen fraction ratio of <300, or lung infiltrates within 24–48 h of >50%. Patients who presented with life-threatening conditions such as respiratory failure, sepsis, or multiple organ dysfunction were also subjected to ICU admission.

Data from the KFHU were collected according to the trial protocol. Two research assistants recorded the data independently. Disagreements were resolved by rechecking the medical records of participants. The data included patient demographics, clinical characteristics, laboratory findings, management and treatment interventions, complications, and outcomes. The main outcome related to our study was discharge status from the ICU (discharged alive or dead). Some of the outcome data were followed up after the recruitment period. All recruited patients with COVID-19 in the ICU of the KFHU were managed according to the Saudi Ministry of Health and the Saudi Critical Care Society Clinical Practice Guidelines. These guidelines were developed by experts in critical care medicine, respiratory therapy, infection control, nursing, and public health and were based on the best available evidence and aligned with international guidelines [16]. The included patients were categorized as Saudis and non-Saudis. Saudi citizens are those who have a Saudi national identification number, whereas non-Saudi residents are those who do not have one.

### 2.4. Statistical Analysis

Patient characteristics and outcomes were reported using descriptive statistics. Continuous variables were reported as the means and standard deviations (SDs) or medians and interquartile ranges (IQRs) as appropriate. Categorical variables were reported as counts and percentages. We stratified the population into Saudi and non-Saudi populations to assess differences in characteristics and outcomes using Student’s *t*-test, the Mann–Whitney U test, and the X^2^ test, as appropriate.

Predictors of mortality across the overall population were assessed using multivariable logistic regression. The variables included were age, sex, SpO_2_%, respiratory rate, comorbidities, intubation, medication therapy, and ICU length of stay. Since more than 15% of the values for some variables were missing at random, we performed multiple imputation. Analyses of complete and imputed data were performed. Effect sizes were expressed as odds ratios (ORs) with 95% confidence intervals (95% CIs). A two-tailed *p* value of less than 0.05 was considered statistically significant. All the statistical analyses were performed using STATA software version 16.0 (Statacorp, College Station, TX, USA).

## 3. Results

After the trial recruitment period and follow-up of outcome data, 185 ICU patients from the KFHU were included. Of those, 90 (48.6%) were Saudis, and 95 (51.4%) were non-Saudis.

### 3.1. General Characteristics

General characteristics across the overall population and in Saudi and non-Saudi populations are presented in Table 1. The mean age across the overall population was 56.7 years, and 71.9% of the population was male. A total of 68 percent of the patients presented with a temperature greater than 38 °C, and 77.3% presented with dyspnea. The overall median respiratory rate was 26.0 breaths/min (IQR: 22.0, 32.0), and the median percentage of SpO_2_ was 89.0% (IQR: 83.0%, 94.0%). The majority of patients had a history of diabetes (54.1%), and 42.7% had hypertension. Compared with Saudis, non-Saudis were younger, with a mean age of 54.4 years, and were more likely to be males (81.1%). The median respiratory rate was significantly greater, and the percentage of SpO_2_ was lower in non-Saudis than in Saudis (both *p* values < 0.001).

### 3.2. Clinical Characteristics

Table 2 presents the clinical characteristics across the overall population and in Saudi and non-Saudi populations. The overall median percentages of lymphocytes and neutrophils were 14.2 (IQR: 9.3, 20.8) and 76.8 (IQR: 70.2, 83.8), respectively. The median D-dimer was 1.3 per µg/mL (IQR: 0.7, 2.4) and 650.0 per µg/L (IQR: 320.0, 1490.0) for ferritin. Evidence of bacterial infection in the respiratory system was found in 20.5% of the patients. More than 70.0% of the patients received hydroxychloroquine, azithromycin, steroids, or antiviral therapy. One hundred eight patients (59.3%) were intubated, and 86 (47.8%) received high-flow nasal oxygenation therapy. Few patients developed complications in the ICU (*n* ≤ 4), and the median length of stay per day in the ICU was 9.0 (IQR: 5.0, 18.0). The laboratory findings differed significantly between Saudis and non-Saudis. Non-Saudis had higher median levels of lactate dehydrogenase (LDH), alanine aminotransferase (ALT), and aspartate aminotransferase (AST) per U/L and ferritin per µg/L.

### 3.3. Discharge Outcomes

**Figure 1:**
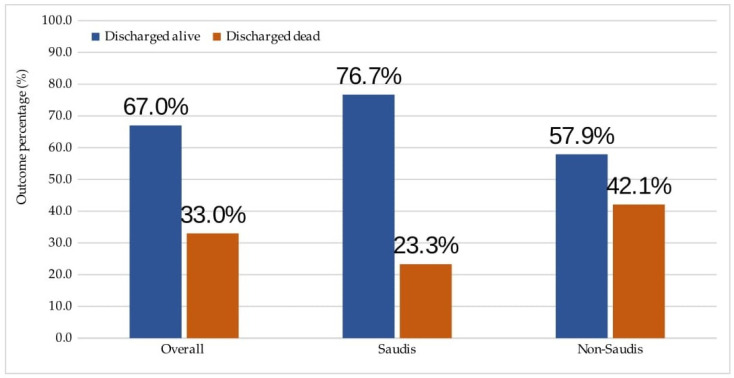
Discharge outcomes from the ICU across the overall population (*n* = 185) and in Saudis (*n* = 90) and non-Saudis (*n* = 95). Discharge outcomes significantly differ between Saudis and non-Saudis (*p* = 0.007).

Figure 1 shows discharge outcomes across the overall population and in Saudi and non-Saudi populations. Among the 185 patients, 124 (67.0%) were discharged alive from the ICU, and 61 (33.0%) died. The mortality rate in non-Saudi populations was twice as high as that in Saudi populations, with a rate of 42.1% compared with 23.3% (*p* = 0.007).

### 3.4. Pridectors of Mortality

Table S1 (Supplementary Materials) presents the univariable analysis of predictors of mortality. Among these predictors, five remained significantly associated with mortality in the multivariable logistic regression model, as shown in Table 3. The OR of mortality was 2.66 (95% CI: 1.05, 6.72) in non-Saudis compared with Saudis, 2.78 (95% CI: 1.04, 7.38) in diabetic patients compared with nondiabetic patients, 0.26 (95% CI: 0.07, 0.92) in patients who had bacterial infection in the respiratory system compared with those who had not, and 8.55 (95% CI: 2.92, 24.97) in intubated patients compared with nonintubated patients. The OR of mortality increased by a factor of 1.0 for a 100 unit per µg/L increase in ferritin (OR 1.01; 95% CI: 1.00, 1.02). Compared with complete data analysis, findings from multivariable logistic regression of imputed data, as shown in Table 4, were nearly identical except for patients with diabetes relative to those without diabetes (OR 2.14; 95% CI: 0.95, 4.84).

## 4. Discussion

During the first wave, the overall mortality rate of COVID-19 patients in the ICU was 33.0%. The mortality rate in non-Saudis (42.1%) was nearly twofold greater than that in Saudis (23.3%). In the non-Saudi group relative to the Saudi group, some of the initial symptoms and laboratory findings substantially differed. Non-Saudis were more likely to be tachypneic, present with low oxygen saturation, have low levels of lymphocytes, and have high levels of LDH, ALT, AST, and ferritin. Non-Saudi, ferritin, intubation, and bacterial infection of the respiratory system were associated with ICU mortality.

Our overall rate of mortality from COVID-19 in the ICU during the first wave was comparable to the global rate. In a 2021 systematic review comprising more than 43,000 ICU patients, the global rate was estimated at 35.5% [17]. The Eastern Mediterranean mortality rate in that study was 61.9%, and only four studies were included. These countries included patients from Yemen, Kuwait, Israel, and Iran. A 2020 study from a single tertiary hospital in Riyadh, Saudi Arabia, reported a mortality rate of 25.3%. In that report, the majority of the included population was Saudis (77.3%) [18]. Another study carried out between April and June 2020, comprising data from five tertiary hospitals of a private healthcare corporation in Riyadh, Saudi Arabia, reported a mortality rate of 58.1% [19]. However, participants were recruited without informed consent in that study. In our study, participants were recruited on the basis of informed consent. Patients who have provided informed consent could be healthier.

The difference in mortality rates between Saudis and non-Saudis was remarkable. However, our findings suggest that non-Saudis, relative to Saudis, presented to the hospital with severe symptoms and had poorer laboratory results during the first wave. While racial disparities in healthcare are well documented across many regions [20,21,22,23], this may not necessarily be the case in Saudi Arabia. In our region, all Saudi citizens and non-Saudi residents of legal or illegal immigration status had full access to healthcare facilities since 30 March 2020, including government and nongovernment hospitals [24]. This also included free-of-charge testing, quarantine accommodation, and provision of care in primary and critical care settings. Rather, this difference may be related to disparities in social determinants of health. The evidence indicates that the impact of social determinants of health on population health is greater than that of healthcare [25,26,27,28]. Compared with Saudis, non-Saudis are more likely to have lower income, less access to social support, and more language barriers [29].

Our study revealed that non-Saudi status, ferritin, intubation, and evidence of bacterial infection in the lungs were significant predictors of mortality. Ethnic minorities in many regions are often at greater risk of death from COVID-19 than ethnic majorities [30,31,32]. For example, the risk of death from COVID-19 between March 2020 and February 2021 in the United States was 1.6–7.2 times greater in Asian or Pacific Islander, Black, Latino, and American Indian or Alaskan Native populations than in the White population [32]. Additionally, elevated levels of ferritin in earlier studies were associated with poor prognosis in patients with COVID-19 [5,33,34]. According to a 2021 systematic review, the level of ferritin in patients with COVID-19 was greater in non-survivors than in survivors, with a mean difference of 912.1 (95% CI: 705.2, 1119.0; *p* < 0.001) [34]. Moreover, patients with COVID-19 were at increased risk of death after intubation [35]. Increased age, ferritin levels, and a past medical history of chronic kidney disease were significant predictors of mortality following intubation in COVID-19 patients. The mortality rate was also 15.0% greater in COVID-19 patients who received early intubation over noninvasive ventilation (NIV) than in those who received only NIV [36]. Bacterial infection is also a significant predictor of mortality in COVID-19 patients [37]. Secondary bacterial infection was associated with an increased risk of mortality. However, our findings showed that bacterial infection in the respiratory system was associated with a reduced risk of mortality. This could be explained by the fact that the majority of our patients received antibiotic (86.0%) and antiviral (91.0%) therapy and that our investigated population may have responded well to such therapies.

Our study has important implications. Since the death rate in non-Saudis was higher than that in Saudis during the first wave despite government support in healthcare for both populations, more efforts should be made to improve social determinants of health for non-Saudis. This includes improving their economic stability, neighborhood and physical environment, and community engagement. Before this, qualitative work should be carried out with non-Saudis to guide government initiatives.

Our findings should be interpreted in the context of their limitations. First, our data date back to 2020, when much was unknown about COVID-19 and its treatment interventions. Therefore, our findings should be contextualized against findings reported in the initial phase of the pandemic. Second, since our study was a secondary data analysis, many variables, such as the number of patients who received noninvasive ventilation, the time of intubation (early or delayed), the duration of mechanical ventilation, the Sequential Organ Failure Assessment (SOFA) score, and secondary infection, were not recorded. As such, it is not clear whether these variables affect our mortality rate or predictors of mortality. Other nonmedical variables that could be related to mortality, including income, education, employment status, working life conditions, health insurance, and social inclusion, were also not recorded. Third, our patients were recruited, which may have led to underestimation or overestimation of the mortality rate. Finally, diabetes is a known independent predictor of COVID-19-related mortality [38,39]. However, it was not included in our imputed data. The reason for this could be related to the lack of statistical power in our regression model. The number of included deaths in our model was 61.

## 5. Conclusions

The overall ICU mortality rate of patients with COVID-19 was comparable to the global mortality rate during the initial phase of the pandemic. However, the rate of mortality was twice as high in non-Saudis (42.1%) than in Saudis (23.3%). While both populations had similar access to healthcare during the pandemic, the social determinants of health were likely different between the two groups. Understanding the social determinants of health across minority populations is important for guiding government initiatives.

## Figures and Tables

**Table 1:** General characteristics across the overall population and among Saudis compared with non-Saudis.

	Overall *n* = 185	Saudi *n* = 90	Non-Saudi *n* = 95	*p* Value	Missing, *n* = (Overall, Saudi, Non-Saudi)
Demographics					
Mean age, (SD)	56.7 (13.5)	59.2 (15.6)	54.4 (10.8)	0.016	0
Male gender, *n* (%)	133 (71.9)	56 (62.2)	77 (81.1)	0.004	0
Initial symptoms					
Temperature > 38 °C, *n* (%)	126 (68.1)	62 (68.9)	64 (67.4)	0.824	0
Dyspnea, *n* (%)	143 (77.3)	67 (74.4)	76 (80.0)	0.367	0
Median RR/min, (IQR)	26.0 (22.0, 32.0)	24.0 (21.0, 29.5)	28.0 (23.0, 36.0)	<0.001	(2, 2, 0)
Median SpO_2_%, (IQR)	89.0 (83.0, 94.0)	91.0 (88.0, 96.0)	86.0 (80.0, 91.0)	<0.001	(2, 2, 0)
Dry cough, *n* (%)	114 (61.6)	57 (63.3)	57 (60.0)	0.641	0
Flu symptoms, *n* (%)	5 (2.7)	2 (2.2)	3 (3.2)	0.695	0
Headache, *n* (%)	8 (4.3)	6 (6.7)	2 (2.1)	0.127	0
Diarrhea, *n* (%)	18 (9.7)	13 (14.4)	5 (5.3)	0.035	0
Other symptoms, *n* (%)	78 (42.2)	38 (42.2)	40 (42.1)	0.987	0
Previous medical history, *n* (%)					
Diabetes	100 (54.1)	55 (61.1)	45 (47.4)	0.061	0
Hypertension	79 (42.7)	46 (51.1)	33 (34.7)	0.024	0
Cardiac disease	29 (15.7)	21 (23.3)	8 (8.4)	0.005	0
Liver disease	4 (2.2)	1 (1.1)	3 (3.2)	0.339	0
Renal disease	10 (5.4)	7 (7.8)	3 (3.2)	0.165	0
Autoimmune disease	2 (1.1)	2 (2.2)	0.0 (0.0)	0.144	0
Vitamin D deficiency	2 (1.1)	1 (1.1)	1 (1.1)	0.969	0
Other comorbidities	66 (35.7)	48 (53.3)	18 (19.0)	<0.001	0
Other history, *n* (%)					
Smoker	7 (3.8)	2 (2.2)	5 (5.3)	0.279	0
Obese	8 (4.3)	7 (7.8)	1 (1.1)	0.025	0

Abbreviations: SD, standard deviation; RR, respiratory rate; IQR, interquartile range; SpO_2_, blood oxygen saturation.

**Table 2:** Clinical characteristics across the overall population and in Saudis and non-Saudis.

	Overall *n* = 185	Saudi *n* = 90	Non-Saudi *n* = 95	*p* Value	Missing, *n* = (Overall, Saudi, Non-Saudi)
Baseline laboratory results, median (IQR)					
Lymphocytes %	14.2 (9.3, 20.8)	16.0 (11.1, 21.9)	12.3 (7.4, 20.3)	0.011	(11, 6, 5)
Neutrophils %	76.8 (70.3, 83.8)	75.8 (67.4, 81.1)	78.7 (71.3, 85.7)	0.031	(23, 9, 14)
Albumin per g/dL	3.5 (3.3, 3.8)	3.4 (3.3, 3.7)	3.6 (3.4, 3.8)	0.006	(3, 2, 1)
LDH per U/L	441.5 (346.5, 644.5)	407.0 (321.0, 530.0)	549.0 (403.0, 735.0)	<0.001	(5, 4, 1)
ALT per U/L	34.5 (23.0, 54.5)	28.5 (19.0, 52.0)	40.0 (27.0, 58.0)	0.009	(5, 4, 1)
AST per U/L	43.0 (29.0, 85.5)	33.5 (22.0, 61.0)	50.0 (35.0, 74.0)	<0.001	(5, 4, 1)
CRP per mg/L	10.8 (6.0, 19.0)	9.5 (6.0, 18.6)	11.7 (5.8, 19.5)	0.501	(25, 10, 15)
D-Dimer per µg/ml	1.3 (0.7, 2.4)	1.3 (0.7, 2.7)	1.2 (0.7, 2.0)	0.694	(37, 19, 18)
Ferritin^/100^ per µg/L ^a^	6.5 (3.2, 14.9)	5.5 (2.2, 11.3)	8.2 (4.5, 20.8)	0.013	(52, 26, 26)
Troponin per ng/mL	0.01 (0.01, 0.04)	0.02 (0.01, 0.08)	0.011 (0.01, 0.02)	0.021	(70, 33, 37)
Bacterial infection, *n* (%)					
Blood	21 (11.4)	9 (10.0)	12 (12.6)	0.573	0
Respiratory	38 (20.5)	20 (22.2)	18 (18.9)	0.582	0
Medication therapy, *n* (%)					
Hydroxychloroquine	136 (73.5)	61 (67.8)	75 (78.9)	0.085	0
Azithromycin	159 (85.9)	76 (84.4)	83 (87.4)	0.567	0
Steroids ^b^	135 (73.0)	72 (80.0)	63 (66.3)	0.036	0
Tocilizumab	35 (18.9)	22 (24.4)	13 (13.7)	0.062	0
Antiviral therapy ^c^	168 (90.8)	82 (91.1)	86 (90.5)	0.891	0
ACE II inhibitors	20 (10.8)	12 (13.3)	8 (8,4)	0.282	0
Other medications	176 (95.1)	86 (95.6)	90 (94.7)	0.796	0
Interventions at ICU, *n* (%)					
Intubation ^d^	108 (59.3)	48 (53.9)	60 (64.5)	0.146	(3, 1, 2)
HFNOT	86 (47.8)	45 (51.7)	41 (44.1)	0.305	(5, 3, 2)
Complications at ICU, *n* (%)					
Respiratory insufficiency	3 (1.6)	2 (2.2)	1 (1.1)	0.529	0
Acute kidney injury	4 (2.2)	1 (1.1)	3 (3.2)	0.339	0
ACS	2 (1.1)	1 (1.1)	1 (1.1)	0.969	0
ARDS	1 (0.5)	0	1 (1.1)	0.329	0
Sepsis	1 (0.5)	0	1 (1.1)	0.329	0
Median length of stay in ICU per day, (IQR)	9.0 (5.0, 18.0)	10.5 (4.0, 19.0)	9.0 (6.0, 18.0)	0.901	0

^a^ Ferritin values were divided by 100 for easier presentation of the data. ^b^ Dexamethasone 6–12 mg via intravenous line daily for a total of 10 days. ^c^ Antiviral therapy included oseltamivir, ribavirin, ritonavir/lopinavir, and favipiravir. ^d^ Intubated cases were mechanically ventilated with lung protective strategies (low tidal volume ventilation). Abbreviations: IQR, interquartile range; LDH, lactate dehydrogenase; ALT, alanine aminotransferase; AST, aspartate aminotransferase; CRP, C-reactive protein; HFNOT, high-flow nasal therapy; ACS, acute coronary syndrome; ARDS, acute respiratory distress syndrome; ICU, intensive care unit.

**Table 3:** Predictors of mortality (complete data analysis).

Predictors	OR (95% CI)	*p* Value
Age	1.01 (0.97, 1.05)	0.441
Non-Saudi	2.66 (1.05, 6.72)	0.038
DM	2.78 (1.04, 7.38)	0.040
Ferritin	1.01 (1.00, 1.02)	0.034
Bacterial infection in the respiratory system	0.26 (0.07, 0.92)	0.037
Intubated	8.55 (2.92, 24.97)	<0.001

Abbreviations: OR, odds ratio; CI, confidence intervals; DM, diabetes mellitus.

**Table 4:** Predictors of mortality (imputed data analysis).

Predictors	OR	*p* Value
Age	1.01 (0.98, 1.04)	0.364
Non-Saudi	2.43 (1.12, 5.28)	0.024
DM	2.14 (0.95, 4.84)	0.067
Ferritin	1.01 (1.00, 1.02)	0.043
Bacterial infection in the respiratory system	0.28 (0.10, 0.77)	0.014
Intubated	10.01 (3.98, 25.15)	<0.001

Abbreviations: OR, odds ratio; CI, confidence intervals; DM, diabetes mellitus.

## Data Availability

The dataset analyzed during the current study is available from the corresponding author upon reasonable request.

## Supplementary Materials

The following supporting information can be downloaded at https://www.mdpi.com/article/10.3390/jcm14061915/s1, Table S1: Univariate analysis of predictors of mortality.
