# Supplementary material for: Outcomes of COVID-19 During the First Wave in Saudi Arabia: An Observational Study of ICU Cases from a Single Hospital
Source: J Clin Med. 2025 Mar 12;14(6):1915. doi: 10.3390/jcm14061915 (PMC11942682; doi:10.3390/jcm14061915)
Supplement: Supplementary file 1 [file jcm-14-01915-s001.zip › jcm-3486430-supplementary.pdf]

Table S1. Univariable analysis of predictors of mortality.

	OR	P-value
Demographics		
Age	1.01 (098–1.03)	0.391
Age groups		
< 57 years	Reference	
≥ 57 years	0.93 (0.50, 1.73)	0.840
Gender		
Female	Reference	
Male	2.24 (1.05–4.76)	0.035
Nationality		
Saudi		
Non-Saudi	2.38 (1.26, 4.51)	0.007
Baseline characteristics		
Temperature		
≤ 38 °C	Reference	
> 38 °C	1.68 (0.84–3.35)	0.137
Dyspnoea		
No	Reference	
Yes	0.85 (0.41–1.75)	0.667
Respiratory rate	0.98 (0.94–1.02)	0.369
Blood oxygen saturation (SpO_2%)	0.96 (0.93–1.00)	0.074
Dry cough		
No	Reference	
Yes	0.76 (0.41–1.43)	0.406
Flu symptoms		
No	Reference	
Yes	1.36 (0.22–8.40)	0.736
Headache		
No	Reference	
Yes	0.27 (0.03–2.31)	0.237
Diarrhea		
No	Reference	
Yes	0.22 (0.05–1.03)	0.055
Past medical history		
Diabetes		
No	Reference	
Yes	1.65 (0.88–3.09)	0.116
Hypertension		
No	Reference	
Yes	1.48 (0.79–2.74)	0.213
Cardiac disease		
No	Reference	
Yes	1.08 (0.46–2.49)	0.851
Obesity		
No	Reference	
Yes	0.28 (0.03-2.32)	0.237
Renal disease		
No	Reference	
Yes	2.12 (0.59–7.64)	0.258
Laboratory results		
Lymphocytes	1.00 (0.98–1.02)	0.685
Neutrophils	0.99 (0.97–1.01)	0.444
Albumin	1.05 (0.92–1.19)	0.429
Lactate Dehydrogenase (LDH)	1.01 (1.01–1.01)	0.027
Alanine Aminotransferase (ALT)	1.00 (0.99–1.01)	0.600
Aspartate Aminotransferase (AST)	1.01 (1.00–1.02)	0.050
C-Reactive Protein (CRP)	1.01 (0.98–1.05)	0.261

D-Dimer	1.07 (1.00–1.14)	0.038
Ferritin	1.01 (0.99–1.02)	0.113
Troponin	0.94 (0.80–1.09)	0.427
Evidence of Bacterial infection		
Blood		
No	Reference	
Yes	1.65 (0.57–4.76)	0.347
Respiratory		
No	Reference	
Yes	0.38 (0.16, 0.94)	0.037
Medication therapy		
Hydroxychloroquine		
No	Reference	
Yes	2.34 (1.07, 5.08)	0.032
Azithromycin		
No	Reference	
Yes	0.91 (0.38, 2.19)	0.848
Steroids		
No	Reference	
Yes	0.65 (0.33, 1.28)	0.218
Tocilizumab		
No	Reference	
Yes	0.44 (0.18, 1.08)	0.075
Antiviral therapy		
No		
Yes	1.66 (0.52, 5.35)	0.389
ACE II inhibitors		
No	Reference	
Yes	1.40 (0.54, 3.65)	0.481
Interventions at ICU		
Intubated		
No	Reference	
Yes	7.66 (3.35, 17.48)	<0.001
Received high flow nasal oxygen therapy		
No	Reference	
Yes	0.38 (0.19, 0.73)	0.004
Length of stay in ICU per day	1.00 (0.97, 1.03)	0.674
